# 88. Efficacy of nirmatrelvir-ritonavir in high-risk trial participants with prior SARS-CoV-2 infection or vaccination: a pooled analysis

**DOI:** 10.1093/ofid/ofae631.025

**Published:** 2025-01-29

**Authors:** John M McLaughlin, Heidi Leister-Tebbe, Weihang Bao, Romina Quercia, Jennifer Hammond

**Affiliations:** Pfizer, Collegeville, PA; Pfizer Inc, Collegeville, Pennsylvania; Pfizer Inc, Collegeville, Pennsylvania; Pfizer, Collegeville, PA; Pfizer Inc, Collegeville, Pennsylvania

## Abstract

**Background:**

EPIC-HR, a trial conducted early-on in the COVID-19 pandemic, showed > 85% efficacy of oral nirmatrelvir-ritonavir (NMV/r) for preventing COVID-19 hospitalization and all-cause death in unvaccinated high-risk patients (≥ 1 risk factor for severe COVID-19). The applicability of these findings to current settings of high population-level immunity, however, are not well understood. We evaluated efficacy of NMV/r in a subset of high-risk trial participants with pre-existing SARS-CoV-2 immunity from either prior infection or vaccination.

Efficacy of NMR-r by outcome in high-risk EPIC-HR and EPIC-SR mITT patients with prior SARS-CoV-2 infection or vaccination (n=1600)

**Figure:**
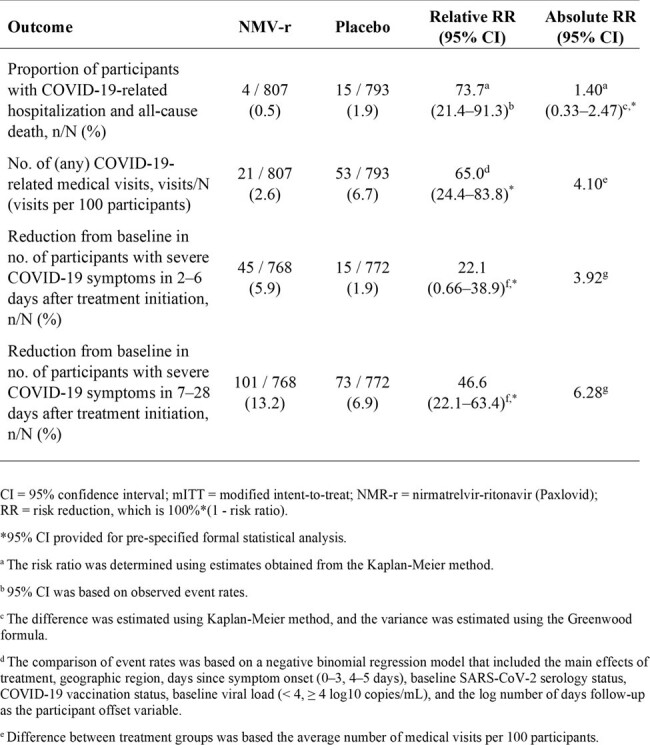

**Methods:**

Efficacy of NMV/r received within 5 days of COVID-19 symptom onset was assessed vs placebo through 28 days against a range of pre-specified outcomes. Data were pooled from two Phase 2/3 RCT modified intention-to-treat populations: (1) high-risk EPIC-HR patients who were unvaccinated and SARS-CoV-2 seropositive at baseline, and (2) high-risk EPIC-SR patients who previously received COVID-19 vaccine.

**Results:**

1600 participants were included, of whom 969 (60.6%) were high-risk and SARS-CoV-2 seropositive from EPIC-HR and 631 (39.4%) were high-risk and vaccinated against COVID-19 from EPIC-SR. Overall, 807/1600 (50.4%) received NMV/r and 793/1600 (49.6%) placebo. Compared to placebo, NMV/r reduced the risk of COVID-19 hospitalization and all-cause death by 73.7% (95% confidence interval: 21.4–91.3%; absolute rate reduction: 1.40% [0.33–2.47%; *P*=.010]), any COVID-19 medical visit by 65.0% (24.4–83.8%; *P*=.008), and having severe COVID-19 symptoms by 22.1% (0.66–38.9%; *P*=.044) and 46.6% (22.1–63.4%; *P*=.001) in the 2–6 and 7–28 days following treatment initiation, respectively (**Table**). Median time to sustained symptom alleviation was 2 days shorter (12 *vs* 14 days; *P*=.047) for those who received NMV/r vs placebo.

**Conclusion:**

In a subset of RCT participants with pre-existing natural or vaccine-derived SARS-CoV-2 immunity, treatment with NMV/r significantly reduced the risk of COVID-19 hospitalization and all-cause death, with a number-needed-to-treat of 71. Treated patients also had fewer COVID-19-related medical encounters, lower risk of having severe COVID-19 symptoms, and shorter time to symptom alleviation. These data underscore the utility of NMV/r in high-risk patients with baseline SARS-CoV-2 immunity.

**Disclosures:**

**John M. McLaughlin, PhD**, Pfizer: Employee|Pfizer: Stocks/Bonds (Public Company) **Heidi Leister-Tebbe, BSN**, Pfizer Inc: Employee|Pfizer Inc: Stocks/Bonds (Public Company) **Weihang Bao, PhD**, Pfizer: Employee|Pfizer: Stocks/Bonds (Public Company) **Romina Quercia, M.D., Ph.D.**, Pfizer: Employee|Pfizer: Stocks/Bonds (Public Company) **Jennifer Hammond, PhD**, Pfizer, Inc: Employee|Pfizer, Inc: Stocks/Bonds (Public Company)

